# O8-8 Foundation of Move Healthy: athletic skill development in children from a motor learning perspective

**DOI:** 10.1093/eurpub/ckac094.064

**Published:** 2022-08-29

**Authors:** Anne Benjaminse

**Affiliations:** School of Sportstudies, Hanzehogeschool Groningen, Groningen, The Netherlands

**Keywords:** injury prevention, co-creation, motor skills, sports, health

## Abstract

**Background:**

In the European Union, 6.1 million people are being treated in hospital annually for a sports injury. Of this, 31% of these injuries affect young people (15-24 years). Injury incidence, medical and lost productivity costs can be reduced by promoting a healthy and active lifestyle, where attention is paid to primary injury prevention. However, after two decades of initiatives, traditional injury prevention prevention programs seem effective in the short-term and controlled study settings, but have not decreased long-term injury incidence. One reason for this is the content of the exercises, currently being mainly ?closed and static? exercises. These exercises don't reflect real-world situations where unexpected and automatic movements are required involving complicated motor control adaptations. In addition, physical educators (PE) and trainers/coaches (TC) neither experience current exercises as being context specific and contributing to their training goals. To overcome this implementation gap, the purpose of the MoveHealthy project is to create exercise routines PE and TC can use, where children acquire the ability to sustain optimal automatic motor control while engaging in complex athletic, unpredictable environments (e.g. movement of another child, or a ball), whilst minimising their risk to sustain an injury.

**Methods:**

Exercise routines (3 for primary and 3 for secondary education, 3 for soccer and 3 for basketball) were co-created with the end-users (PE & TC) and their wishes and needs have been incorporated. Furthermore, to train the complex task-person-environmental interaction, real-world aspects such as visual-motor control where quickly processing environmental cues and anticipation and decision is crucial, was included into all conceptual considerations.

**Results:**

Twelve prototype exercises have been developed. Merging theoretical foundations of motor learning and wishes and needs of end-users made it possible to create exercises that serve both needs.

**Conclusions:**

The development of these prototype exercises guides towards further validation and final development of innovative exercise routines where real-world aspects are incorporated. With this, we will better ensure real-world effects of injury reduction.

**Acknowledgements:**

This project is financially co-funded by the Erasmus+ Sport program from the European Union.

